# Okra mucilage as an encapsulating agent for magnesium hydroxide nano-capsules in oral drug delivery

**DOI:** 10.1038/s41598-025-06274-5

**Published:** 2025-07-01

**Authors:** Masoud Safari Yazd, Amirreza Shafieeha, Fatemeh Eslami, Leila Zargarzadeh

**Affiliations:** 1 Department of Chemical Engineering, Tarbiat Modares University, Jalal Ale Ahmad Highway, P.O. Box 14115-111, Tehran, Iran; 2 Department of Chemical Engineering, Amirkabir University of Technology (Tehran Polytechnic), No. 424, Hafez Ave, P.O. Box 15875-4413, Tehran, Iran

**Keywords:** Nanoencapsulation, Okra mucilage, Magnesium hydroxide nano-capsules, Oral drug delivery systems, Plant-based encapsulating agents, Controlled release, Nanoscale materials, Chemical engineering

## Abstract

The study investigates the utilization of okra mucilage as an encapsulating agent for the development of magnesium hydroxide nano-capsules for oral drug delivery systems. Given the advancements in drug delivery systems (DDSs) and the emerging interest in nanostructured drug delivery systems (NDDSs), the potential of okra mucilage for nanoencapsulation is explored. NDDSs hold promise for enhancing therapeutic efficacy while minimizing adverse effects. Okra mucilage is known for its biodegradability, non-toxicity, and cost-effectiveness, making it a suitable candidate for encapsulation processes. The sol–gel encapsulation method is employed to fabricate the encapsulated magnesium hydroxide particles (EMgPs). The EMgPs were characterized using XRD, FT-IR, Raman spectroscopy, and FESEM/EDS, confirming the successful encapsulation of magnesium hydroxide within the okra mucilage. The hydrophilic properties of the EMgPs were also assessed through contact angle measurements, revealing promising wettability for efficient drug release in the digestive system. Release tests in a simulated digestive system environment demonstrated a controlled and sustained release profile (zero-order release) of magnesium hydroxide from the EMgPs with a rate constant of 0.75 and 0.2894 mg mL^−1^ h^−1^ in gastric phase and intestinal phase, respectively. The findings highlight the potential of okra mucilage as an encapsulating agent in oral drug delivery systems and provide insights for further research in the field of nanomedicine.

## Introduction

Over the past decade, significant research has been directed towards advancing drug delivery systems (DDSs) to enhance therapeutic outcomes while minimizing undesirable side effects. Since the initial approval of DDS by the FDA in 1990, various types of DDS, including nanoparticles (NPs), microspheres, and hydrogels, have been developed to improve drug solubility, stability, and targeted delivery^1^.

Among these innovations, nanostructured drug delivery systems (NDDSs) have gained prominence due to encapsulate, trap, or combine therapeutic compounds within nanoscale carriers. NPs, typically ranging from 1 to 100 nm, exhibit unique properties and can be derived from various materials, including polymers, lipids, and metals, making them suitable for a wide range of biomedical applications^2,3^. NDDSs offer several advantages over conventional systems, such as prolonged circulation time, improved pharmacokinetics, minimized side effects, and the ability to overcome physiological barriers. Despite these benefits, NDDSs still face challenges such as inadequate drug loading, rapid release, poor tissue distribution, and potential toxicity. To address these limitations, researchers have developed strategies such as nanoencapsulation—a technique that coats active pharmaceutical ingredients with polymeric or lipid-based materials to enhance stability and enabled controlled release^1–3^.

Key goal in drug delivery design is to achieve zero-order release, where the drug is released at a constant rate, regardless of its concentration. This mechanism is considered ideal because it maintains consistent therapeutic levels over extended periods, reducing the frequency of dosing and minimizing fluctuations in drug concentration. McHugh et al.^4^ highlight zero-order release as a promising approach to improve therapeutic outcomes and enhance patient adherence. While numerous strategies have been proposed to achieve this, encapsulation using hydrogels and biodegradable polymers such as PLGA (poly(lactic-co-glycolic acid)), remains among the most effective methods due to their biocompatibility and customizable release profiles^5–10^.

Biopolymers like alginate are widely used in encapsulation due to their biocompatibility, gel-forming ability, and capacity to protect drugs from degradation, particularly in the gastrointestinal (GI) tract. Alginate-based encapsulation systems enhance the oral bioavailability of poorly soluble and lipophilic drugs by protecting them from oxidation and enzymatic degradation^11^. Similarly, plant-derived materials, particularly mucilages, have gained interest due to their natural origin, safety, and multifunctionality.

Mucilages are polysaccharide-rich hydrocolloids that vary in chemical composition and functionality. Among these, okra (*Abelmoschus esculentus*) mucilage has emerged as a promising encapsulating material for pharmaceutical and nutraceutical applications. It possesses favorable characteristics such as high biocompatibility, non-toxicity, biodegradability, and low cost of production. Beyond its conventional functions as a binding, thickening, and stabilizing agent, okra mucilage exhibits several health-promoting properties including antimicrobial, antihypertensive, antioxidant, antiasthmatic, and hypoglycemic effects^12–14^. It has also shown compatibility with various bioactive compounds, such as vitamins, and minerals, making it suitable for encapsulating purposes^15–17^. It has also demonstrated efficacy in protecting probiotic cells during their passage through the GI tract, often outperforming traditional materials like sodium alginate^18^. The versatility of okra mucilage enables its use in various encapsulation techniques, including spray drying, coacervation, and emulsification, providing flexibility for different application needs^19–21^.

In this study, we apply okra mucilage as a natural encapsulating material for magnesium hydroxide, an inorganic compound widely used for its antacid and laxative properties. Magnesium hydroxide is commonly formulated as a syrup to neutralize gastric acid and relieve indigestion and constipation^22–26^. However, unlike many organic drugs, it faces formulation challenges such as poor stability, limited shelf life in liquid form, and dosing difficulties. These issues make it an ideal model drug to evaluate the protective and controlled-release capabilities of okra mucilage.

Using magnesium hydroxide also enables exploration of encapsulation strategies for inorganic drugs, which are less commonly studied than their organic counterparts. This expands the application potential of natural polymer-based delivery systems and lays groundwork for future studies on diverse drug types. Encapsulating magnesium hydroxide in a dry dosage form using okra mucilage offer multiple advantages: The mucilage can form a protective barrier around the active ingredient, enhancing stability, extending shelf life, and enabling controlled release in the GI tract. Additionally, converting the formulation from liquid to a dry form improves ease of administration, dosing convenience, and transportability, benefits that are particularly valuable in pediatric and geriatric settings. Moreover, the functional properties of okra mucilage may provide synergistic health benefits, such as antioxidant and antimicrobial activity, which can enhance the overall therapeutic effect^27–29^.

## Material and methods

### Material

Fresh okra was purchased from a local market. The analytical grade of distilled water, ethanol (96%), sodium chloride (99.5%), potassium chloride (99.5%), calcium chloride (98%), sodium carbonate (99.9%), hydrochloric acid (37%), magnesium hydroxide (95%), and pepsin enzyme (from porcine gastric mucosa) 0.7 FIP-U/mg were supplied from Merck Company.

### Mucilage preparation

In order to fabricate the magnesium hydroxide nano-capsules, the encapsulating agent, which is okra mucilage, must be prepared. Firstly, an efficient procedure for extracting okra mucilage needs to be considered, one that is not only well-organized but also yields the maximum output^30^.

Fresh okras dry by being exposed to a hot air stream at 40°C for 48 h in a dryer. Then, the dried okras are powdered. In accordance with another study^31^, to promote mucilage extraction from plants, it is suggested to powder them before beginning the extraction treatment. 15 g of powdered okra is added to 100 mL of water and mixed on a magnetic stirrer at 50°C for 1 h^32^. In the next step, a centrifuge is applied to separate the liquid phase, which includes the proposed mucilage, from the waste cake. Through this procedure, approximately 30 mL of mucilage is obtained. Next, the mucilage is washed with ethanol to precipitate the carbohydrate string and remove pigments from it^31^. In accordance with other studies^31,33–35^ the majority of okra’s chemical composition and its sedimentation string consist of carbohydrates. Ethanol is added slowly, drop by drop, to the mucilage, twice its volume, while mixing with a magnetic stirrer. Consequently, an interconnected and fibrous tissue is precipitated, which should be separated and dried in an oven at 70°C under vacuum for 12 h. Eventually, the precipitated tissue is powdered.

### Encapsulated magnesium hydroxide particle fabrication

**Fig. 1:**
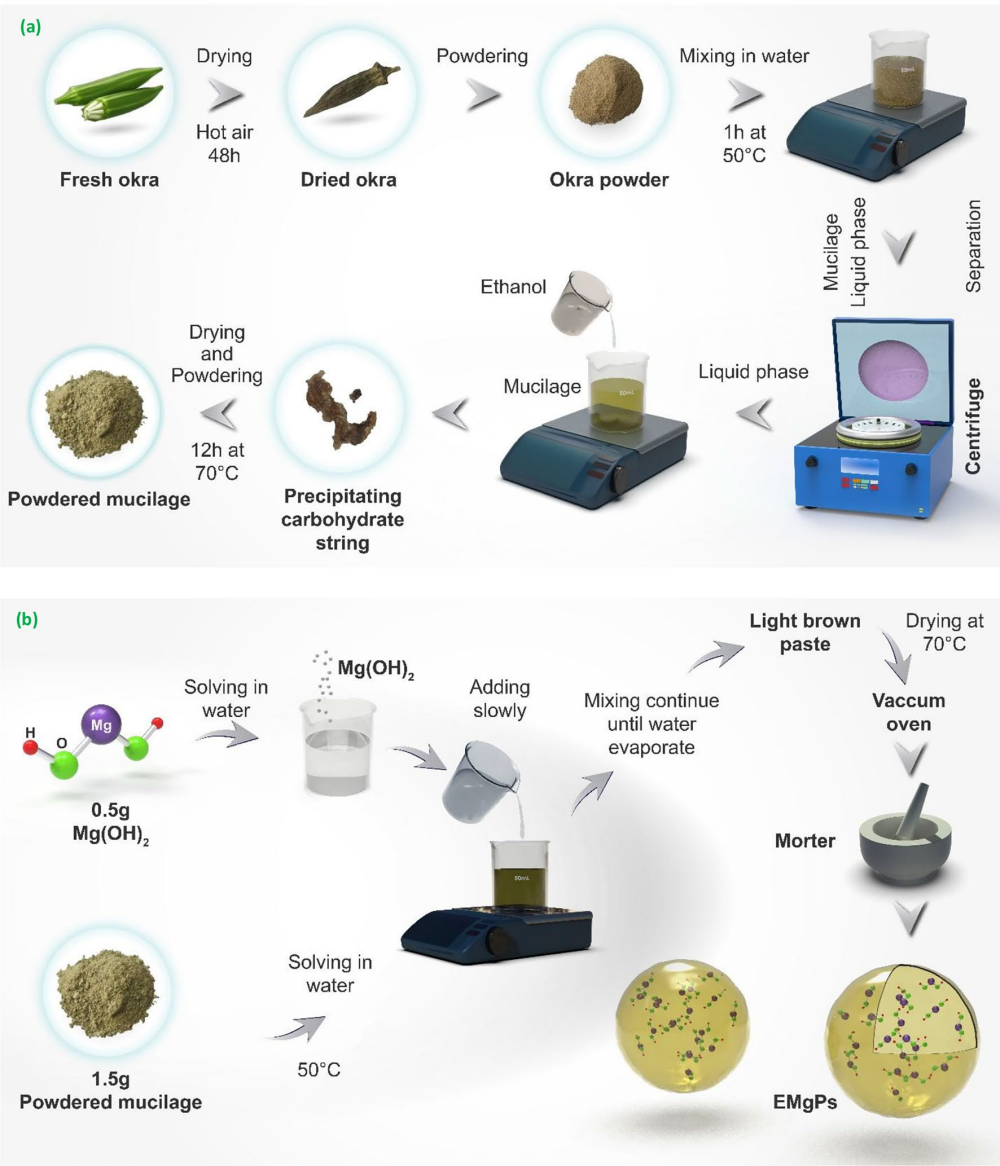
Mucilage preparation (**a**) and EMgPs fabrication (**b**).

Encapsulation is a method that can be physical, chemical, or mechanical, where the core material is enclosed within a covering of wall material, resulting in particles that can range in size from a few nanometers to several millimeters^36^. Although there are numerous encapsulation methods, in this study, the sol–gel encapsulation method is employed to encapsulate Mg(OH)_2_ NPs. The process begins with the preparation of okra mucilage powder, which is dissolved in water under vigorous stirring to ensure complete dispersion. Simultaneously, magnesium hydroxide powder is uniformly dispersed in water. The dispersed magnesium mixture is then slowly added to the mucilage mixture while maintaining continuous stirring to ensure uniform distribution. The weight ratio of mucilage powder to Mg(OH)_2_ powder is maintained at 3:1. The mixture of mucilage and Mg(OH)_2_ is then continuously stirred using a magnetic stirrer at 50°C until all the water has evaporated, resulting in a thick paste. This paste is subsequently dried in a vacuum oven at 70°C to remove any remaining moisture and achieve a stable encapsulated product. The entire detailed procedure, from the initial preparation of mucilage to the final encapsulation process, leading to the formation of EMgPs, is illustrated in Fig. 1a and b.

### Characterization methods

Two primary spectroscopic techniques, X-ray Diffraction (XRD) and Fourier-transform infrared (FTIR), are utilized to determine the chemical and structural characteristics of the encapsulated magnesium hydroxide particles (EMgPs). XRD patterns are acquired using a Philips X’Pert MPD X-ray diffractometer employing monochromatic Cu Kα radiation (λ = 0.154060 nm). FTIR absorption spectra are generated using a Nicolet IR 100 spectrometer covering a range from 400 to 4000 cm^−1^. Subsequently, a combined surface analysis approach involving Raman spectroscopy and field emission scanning electron microscopy/energy dispersive X-ray spectroscopy (FE-SEM/EDS) is conducted. Elemental composition mapping via FE-SEM/EDS is performed using a TESCAN MIRA3. Raman spectra are acquired utilizing the Almega Thermo Nicolet Dispersive Raman Spectrometer, covering evaluations from 100 to 4200 cm^−1^. Furthermore, the magnesium hydroxide released in a simulated digestive system solution is quantified using Inductively Coupled Plasma Atomic Emission Spectroscopy (ICP-OES) Varian-735.

### Surface energy determination

To calculate the surface energy of EMgPs, Mg(OH)_2_ and okra mucilage, OW (Owens–Wendt) method is used. First, tablets of each material are prepared, and the contact angles of water and formamide on them are measured. Using the dispersion and specific (polar and hydrogen bonding) contribution of the surface tension of water and formamide, the surface energy of each material is evaluated through Eq. 1^37^:

1 $${\gamma }_{L}\left(1+cos\theta \right)=2\sqrt{{\gamma }_{S}^{d}{\gamma }_{L}^{d}}+2\sqrt{{\gamma }_{S}^{spec}{\gamma }_{L}^{spec}}$$

where the dispersion contribution of surface tension, ${\gamma }_{L}^{d},$ for water and formamide are 21.8 and 39.5 $\frac{mN}{m},$ respectively, and the specific contribution of surface tension, ${\gamma }_{L}^{spec},$ for water and formamide are 51 and 19 $\frac{mN}{m},$ respectively. By solving two equations for each material, the dispersion and specific portion of the material’s surface energy, ${\gamma }_{s},$ can be obtained. The surface energy is the sum of dispersion and specific contributions^37^. The contact angles are measured using a Jikan CAG-10 Contact Angle Goniometer.

### Release procedure for encapsulated magnesium hydroxide particles

First, a suitable biological environment resembling the conditions of the digestive system is prepared to evaluate the release of magnesium hydroxide from the EMgPs sample within it. To achieve this, we dissolve 0.63 g of sodium chloride, 0.229 g of potassium chloride, 0.0229 g of calcium chloride, and 0.12 g of Na_2_CO_3_ in 100 cc of distilled water. The pH of the final solution is adjusted to 2 using an 8N hydrochloric acid solution, mimicking the acidic conditions of the stomach^38^. Then, 0.3% by volume of pepsin, the main digestive enzyme, is added to the medium and thoroughly mixed for 5 min, resulting in a simulated gastric fluid.

**Fig. 2:**
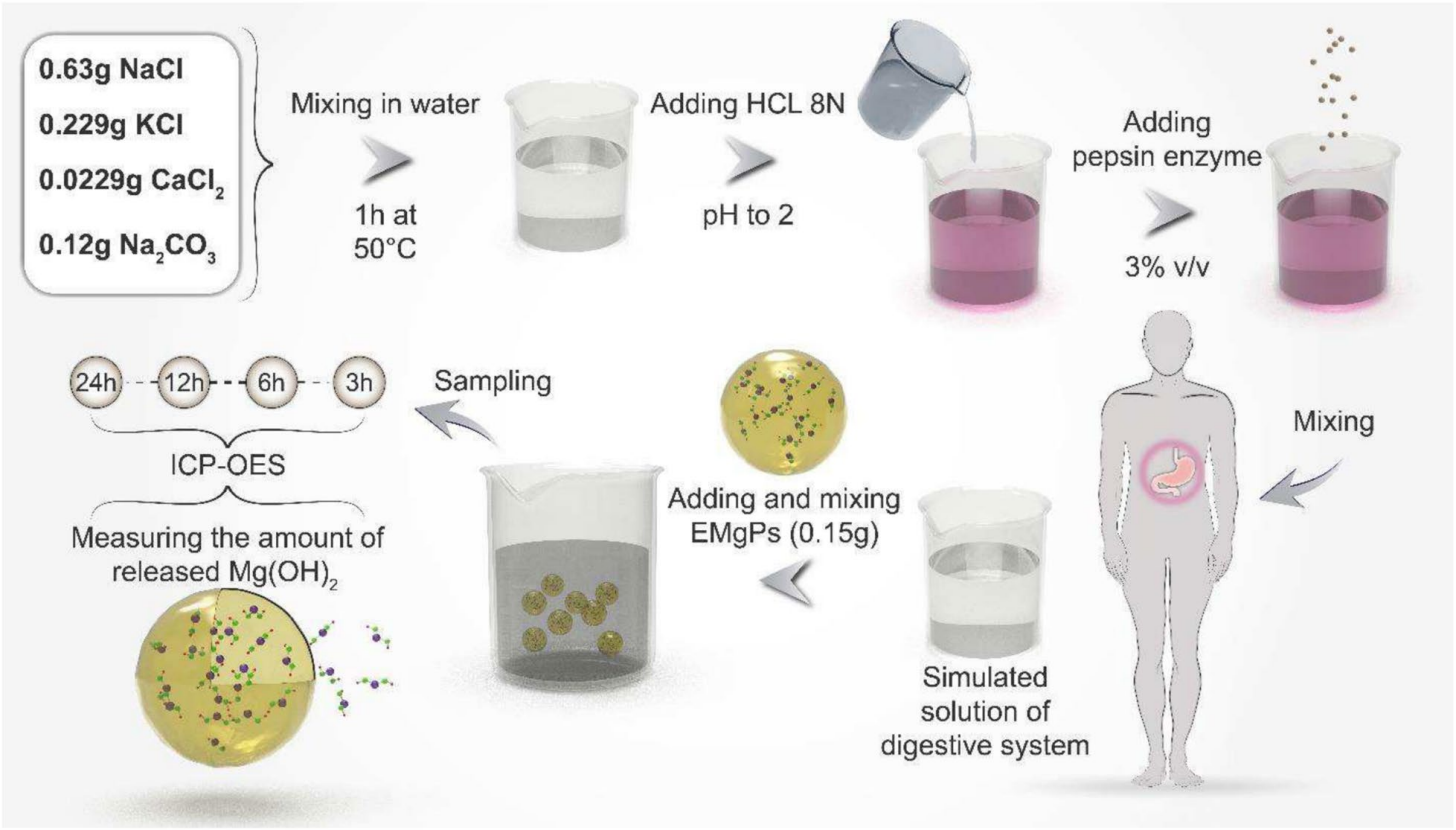
Detailed procedure of Mg(OH)_2_ release test, including the preparation of simulated digestive system solution.

Next, to measure the release of the desired medicinal substance, Mg(OH)_2_, we add 0.15 g of the EMgPs sample into 50 mL of the prepared simulated gastric fluid and stir for 1 min until the particles are completely dispersed. The vessel is then placed in an oven at a temperature of 37°C, replicating human body temperature. At specific intervals of 3, 6, 12, and 24 h, the vessel is placed on a stirrer and stirred at a speed of 500 RPM to ensure uniform distribution of released ions. Subsequently, 1 mL of the sample is taken (in a microtube) and centrifuged. The upper phase is transferred to a falcon tube, and its volume is adjusted to reduce its pH. Using ICP-OES, the amount of released magnesium is accurately measured. The detailed procedure for preparing the simulated solution is displayed in Fig. 2. To validate the effectiveness of EMgPs, we also conducted release tests on Mg(OH)₂ alone—without encapsulation in mucilage —for comparison. A control solution was prepared by adding 50 mg of Mg(OH)₂ to 50 mL of simulated gastric fluid. The same procedure used for measuring the release of EMgPs was applied to the unencapsulated Mg(OH)₂.

In addition, following the gastric phase described above, the samples are transferred to simulated intestinal fluid (SIF) to mimic conditions in the small intestine. The SIF is prepared by dissolving 0.68 g of monobasic potassium phosphate (KH_2_PO_4_) in 100 mL of distilled water, then adjusting the pH to 6.8 using 0.2N NaOH. To simulate enzymatic activity, pancreatin is added at a final concentration of 0.1% w/v. The samples are incubated in this solution at 37°C for an additional 6–8 h with gentle agitation. Aliquots are collected at predetermined time intervals (2 and 4 h) from both the gastric and intestinal media. The release of magnesium ions is quantified using ICP-OES.

## Results and discussion

### Physicochemical characterization of encapsulated magnesium hydroxide particles

Several characterization techniques have been employed to analyze the physicochemical and structural properties of the EMgPs. These techniques provide a comprehensive understanding of the encapsulated magnesium hydroxide and the efficiency of the encapsulation process.

**Fig. 3:**
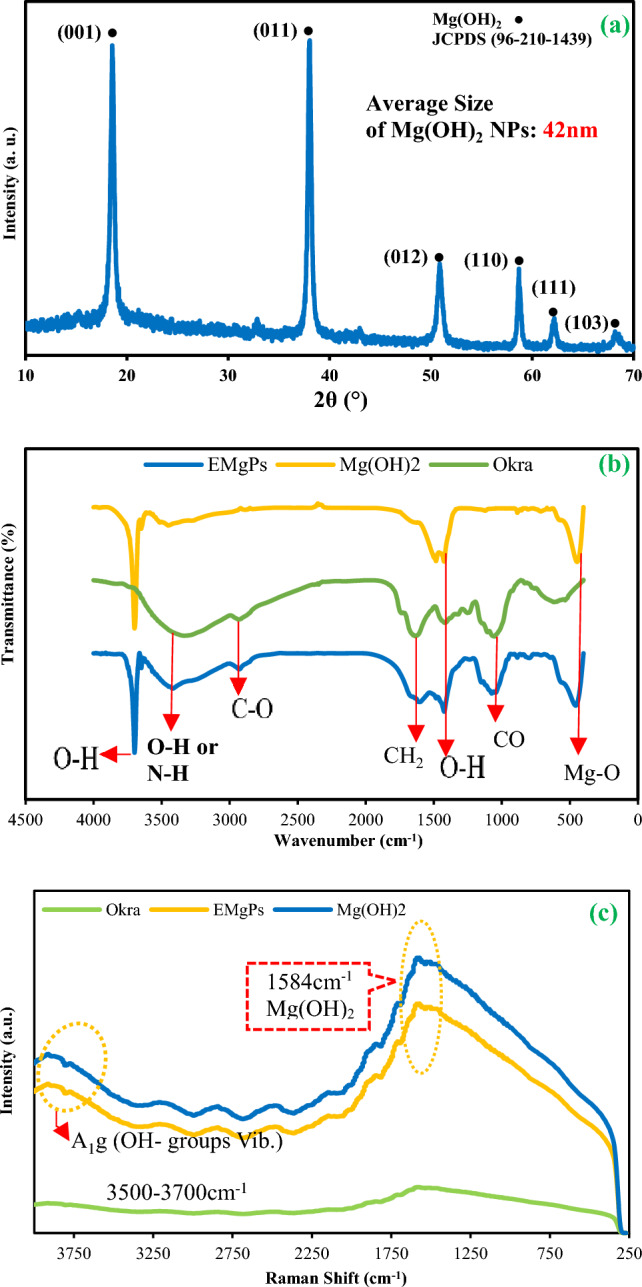
**a** XRD pattern of EMgPs; **b** FT-IR spectra of EMgPs, Mg(OH)_2_ and okra mucilage; **c** Raman spectra of EMgPs, Mg(OH)_2_, and okra mucilage.

In Fig. 3a, XRD patterns are presented, revealing the characteristic peaks of crystalline Mg(OH)_2_ in the EMgPs. The distinct diffraction peaks corresponding to Mg(OH)_2_ in the EMgPs sample are evident at 2θ = 18.55°, 37.97°, 50.77°, 58.6°, 62.03°, and 68.12°. These peaks correspond to the (001), (011), (012), (110), (111), and (103) crystal planes of magnesium hydroxide, respectively, consistent with the standard reference (JCPDS 96–210-1439)^39,40^. The XRD patterns unequivocally demonstrate the presence and complete encapsulation of magnesium hydroxide within the EMgPs sample. The clear and sharp peaks indicate a high degree of crystallinity, suggesting that the encapsulation process did not disrupt the crystalline structure of Mg(OH)_2_. Moreover, Fig. 3a displays the average size of Mg(OH)_2_ NPs, calculated from the XRD patterns using the Scherrer equation (Eq. 2)^41^. The Scherrer equation is a formula that relates the size of crystallites in a solid to the broadening of a peak in a diffraction pattern:

2 $$d=\frac{0.89 \lambda }{\beta \text{ cos}\theta }$$

Here, λ represents the wavelength of the X-ray, while β denotes the width of the peak at half of its maximum height. Using this equation, the average size of the Mg(OH)_2_ NPs is calculated to be 42 nm. This calculated size aligns with the specification data provided by Merck for unprocessed Mg(OH)_2_ NPs (code: 632,309), confirming the successful encapsulation of Mg(OH)_2_ NPs by okra mucilage without any undesirable agglomeration. The agreement between the measured size and the specification data indicates that the encapsulation process using okra mucilage preserves the integrity and size of the Mg(OH)_2_ NPs, preventing them from clustering together. This is crucial because agglomeration can negatively impact the effectiveness of the NPs in their intended applications. The successful encapsulation and preservation of nanoparticle size highlight the potential of okra mucilage as an effective encapsulating agent, ensuring the stability and functionality of the NPs within the EMgPs.

The FTIR spectra of the EMgPs, Mg(OH)_2_, and okra mucilage powder, shown in Fig. 3b, highlight the diverse species present within them. The spectra cover the range of 400–4000 cm^−1^, displaying adsorption bands characteristic of various functional groups and molecular interactions. The observed peak around 3700 cm^−1^ corresponds to the stretching vibrations of hydroxyl groups in the magnesium hydroxide structure, which is consistent across both the EMgPs sample and Mg(OH)_2_ particles spectra^37,38^. A common peak at 3421.6 cm^−1^ is observed in both EMgPs and okra particles, attributed to the capsule wall surrounding the magnesium hydroxide particles formed from okra mucilage^42,43^. This peak is characteristic of O–H stretching or N–H stretching vibrations within the okra mucilage structure, confirming the involvement of this material in forming the encapsulation matrix^44^.

As the spectrum progresses, the asymmetric stretching vibration of hydrocarbons is marked by a peak at 2930.8 cm^−1^, indicative of C-H stretching in aliphatic compounds within the okra mucilage^45^. This confirms the organic nature of the encapsulating agent. Additional peaks at 1603.06 cm^−1^, 1423.7 cm^−1^, and 1073.8 cm^−1^ relate to the CH_2_ bending vibration, hydroxyl group bending vibration, and C-O stretching vibration, respectively. These peaks further validate the carbohydrate components of the okra mucilage, as these functional groups are typical of polysaccharides and other carbohydrate derivatives^46,47^. Furthermore, all these peaks are also observed in the EMgPs, which indicates the presence of okra mucilage in the EMgPs structure.

The spectra also show O–H stretching similar to that in the Mg(OH)_2_ samples, with methylene group bending vibrations visible at 798.9 cm^−1^ and 886.06 cm^−1^, confirming the structural integrity and presence of okra mucilage, albeit weakly. Peaks spanning from 2930 to 886 cm^−1^ highlight the different functional groups in the okra mucilage forming the capsule wall. These functional groups further confirm the carbohydrate characteristics of the EMgPs’ wall, validating the successful integration of okra mucilage as an encapsulating agent crucial for the stability and functionality of the nano-capsules. A significant peak at 458.9 cm^−1^ indicates the presence of magnesium hydroxide, confirming the Mg-O stretching vibrations^48^. This peak, consistent across both encapsulated samples and Mg(OH)_2_ particles, verifies that magnesium hydroxide is successfully integrated within the okra mucilage matrix. Incorporating the third FTIR profile, which overlays the spectra of EMgPs, Mg(OH)_2_, and okra, further substantiates the encapsulation process. The similarities between these spectra, particularly the consistent Mg-O and carbohydrate-related peaks, provide comprehensive evidence that Mg(OH)_2_ is effectively encapsulated by okra mucilage.

Figure 3c depicts the Raman spectra of three samples. Raman spectroscopy is utilized for analyzing crystalline structures and is commonly employed in identifying metal oxides and their compositions due to its sensitivity to vibrational modes of molecules. In the spectra, a broad and intense band observed between 3500 and 3700 cm^−1^ represents the stretching vibrations of hydroxyl groups, corresponding to the A1g vibrational mode^49^. This mode is characteristic of Mg(OH)_2_, reflecting the stretching of O–H bonds. The pure Mg(OH)_2_ sample exhibits a sharper and more intense peak in this region compared to the other samples. The EMgPs spectrum, while also showing peaks in the same hydroxyl region, displays a noticeably reduced intensity compared to the pure Mg(OH)_2_. This suppression is likely due to encapsulation effects of okra mucilage, being largely inert and amorphous, dampens the vibrational signals of the encapsulated core, suggesting successful coating and interaction with the magnesium hydroxide particles. The peak at approximately 1584 cm^−1^ assigned to the vibrational mode of the Mg-OH bond^50^. This peak is clearly present in both Mg(OH)_2_ and EMgPs spectra, with the latter showing it at a slightly lower intensity. This further supports the preservation of Mg(OH)_2_'s structural integrity within the encapsulated form while also indicating the successful embedding of the active compound within the mucilage matrix. In contrast, the spectrum of pure okra mucilage shows minimal Raman activity in both regions, confirming its inert Raman behavior and lack of intrinsic Mg(OH)_2_-related vibrations. This also suggests that any signals observed in the EMgPs spectrum stem from the encapsulated magnesium hydroxide and not the okra itself. Collectively, the Raman results confirm the presence of magnesium hydroxide within the EMgPs (as indicated by characteristic Mg–OH peaks), the retention of hydroxyl functional groups after encapsulation, and the damping effect of okra mucilage on Raman intensity, signifying effective encapsulation and coating. This analysis aligns with the FTIR data and supports the chemical integrity and uniform distribution of Mg(OH)_2_ within the mucilage matrix, which is essential for controlled oral drug delivery.

**Fig. 4:**
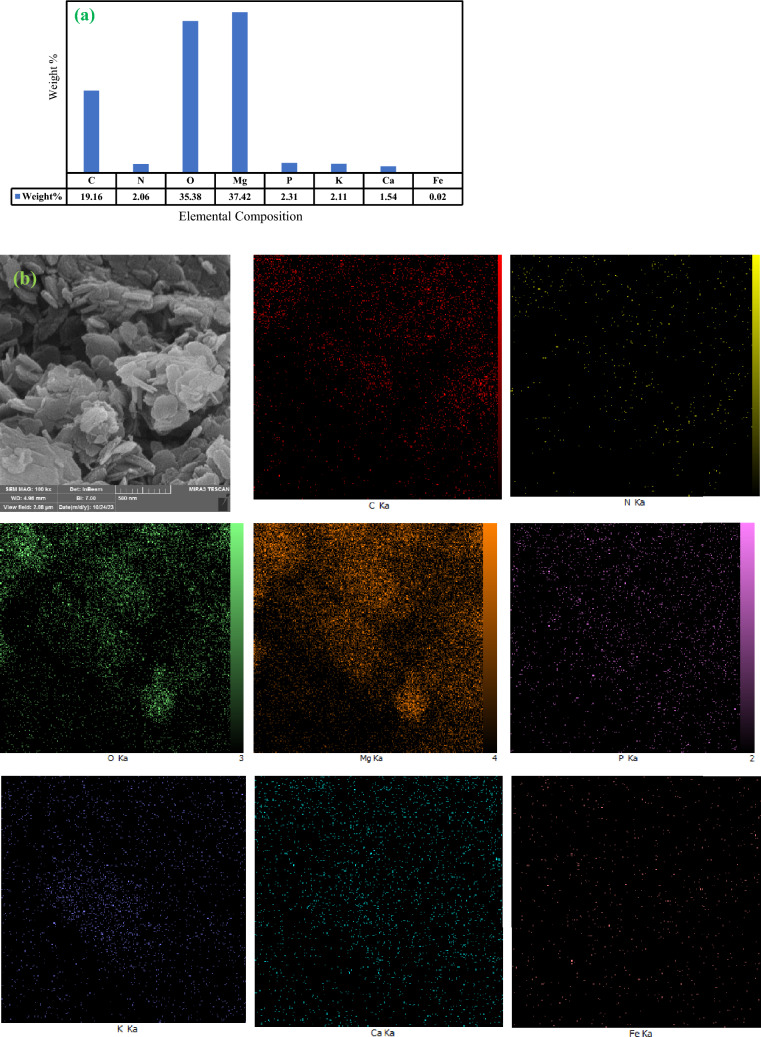
The FESEM/EDS analysis of the elemental composition of EMgPs (**a**), and the FESEM/EDS image and elemental mapping of EMgPs (**b**).

Overall, to conduct a thorough assessment of the elemental composition of EMgPs, FE-SEM/EDS analysis is employed. The chemical composition of the EMgPs sample is illustrated in Fig. 4a, providing significant evidence of the proportion of Mg(OH)_2_ loading into the EMgPs. This analysis not only confirms the presence of magnesium but also quantifies the elemental composition, offering insights into the efficiency of the encapsulation process. Additionally, images of the EMgPs sample and its elemental mapping are presented in Fig. 4b, indicating a uniform distribution of magnesium elements throughout the EMgPs sample. The uniform distribution is crucial for ensuring consistent performance of the nano-capsules in their intended applications. Elemental mapping visually confirms that the magnesium is evenly dispersed within the okra mucilage matrix, further supporting the successful encapsulation process.

Since the hydrophilic properties of the encapsulating agent significantly enhance the efficiency of oral drug nano-capsules by ensuring stable solubility in the digestive system^11,51^, the surface energy of EMgPs was evaluated. To provide further insights, the surface energies of Mg(OH)₂ and okra mucilage were also assessed using the method described in section "Surface energy determination". The results of the contact angle measurements for water and formamide droplets on these surfaces, along with the obtained surface energy of each material, are presented in Table 1.

**Table 1:** Contact angles and surface energies on EMgps, Mg(OH)_2_ and Okra mucilage.

	EMgPs	Mg(OH)_2_	Okra mucilage
Contact angle on water (receding, advancing)	(34.1°, 46.8°)	(36.15°, 40.8°)	(40.8°, 41.1°)
Contact angle on formamide (receding, advancing)	(68.8°, 69.9°)	(24.5°, 24.6°)	(54.1°, 59.6°)
Surface energy (dispersion contribution, specific contribution),$\frac{mN}{m}$	78.3 (0, 78.3)	58.3 (22.4, 35.9)	63.3 (3.3, 60.1)

**Fig. 5:**
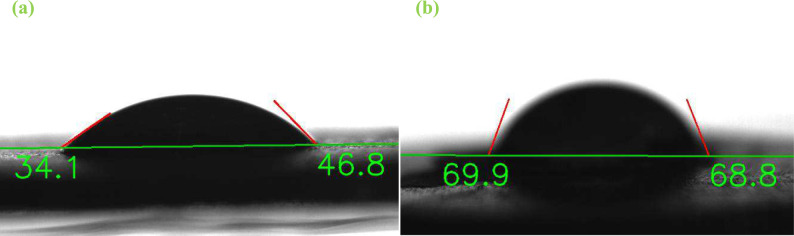
Contact angle of water drop (**a**) and formamide drop (**b**) on the EMgPs’ surface.

Figure 5a and b illustrate the receding and advancing contact angles of formamide and water droplets on the EMgPs surface, respectively. According to section "Surface energy determination", water is a polar material with high non-dispersion interactions like hydrogen-bonding. In contrast, while formamide is also polar, it exhibits higher dispersion interactions. The evaluated surface energies show that synthesized EMgPs are highly hydrophilic with predominantly non-dispersion interactions. Although magnesium hydroxide exhibits moderate dispersion interactions, encapsulating it with okra mucilage results in surface properties similar to okra mucilage.

A lower contact angle of EMgPs with water indicates better wettability and hydrophilicity, while a higher contact angle with formamide suggests a greater affinity for polar materials, characteristic of hydrophilic surfaces. This hydrophilic nature is crucial as it ensures that the nano-capsules can dissolve properly in the aqueous environment of the digestive tract, enhancing the bioavailability and effectiveness of the encapsulated drug. FTIR results confirm the presence of polar functional groups in the EMgPs, supporting their hydrophilic attributes.

### Release profile of encapsulated magnesium hydroxide particles

**Fig. 6:**
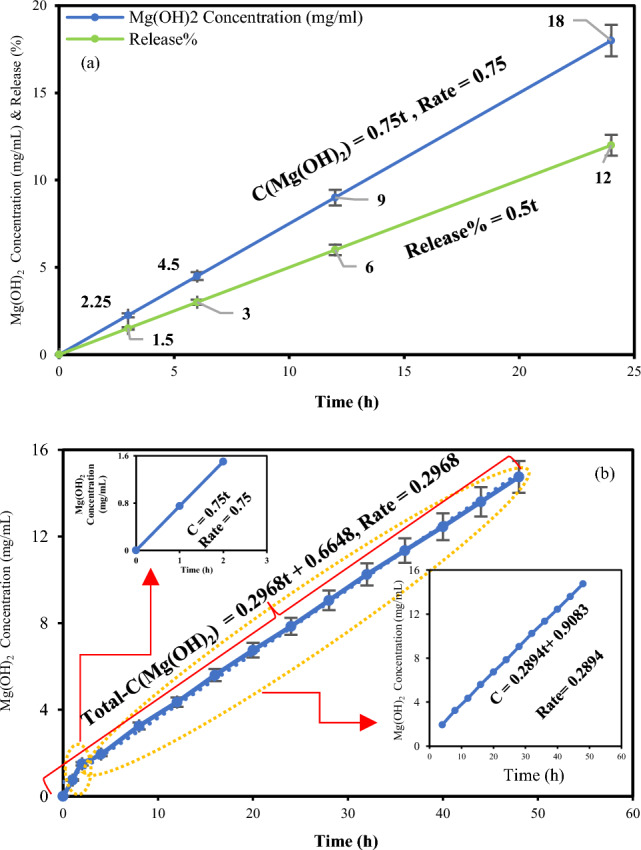
Release profile of Mg(OH)₂ from EMgPs (**a**) in simulated gastric fluid; **b** sequential release in simulated gastric fluid followed by simulated intestinal fluid. Error bars represent the standard deviation from triplicate measurements.

The release of magnesium hydroxide from EMgPs over time in the simulated gastric fluid is depicted in Fig. 6a. The results show a steady and nearly linear increase of magnesium hydroxide in the solution over time, reaching 2.25 mg/mL after 3 h, progressively increasing to 4.5 mg/mL at 6 h, 9 mg/mL at 12 h, and 18 mg/mL at 24 h. The control solution containing only Mg(OH)₂ without mucilage shows complete release at all time intervals, confirming the effectiveness of EMgPs release. Linear regression analysis of cumulative release versus time yielded an R^2^ value of 1, indicating an excellent fit to a zero-order release kinetic model with a rate constant of 0.75 mg mL^−1^ h^−1^. This suggests a controlled and sustained release profile, which is desirable for therapeutic applications. Zero-order release kinetics typically occur in systems where the release process is independent of factors like concentration gradients or solubility, ensuring a constant release rate of Mg(OH)_2_ over time until all of the substance is released from the EMgPs. This characteristic is particularly beneficial in various sustained-release formulations and controlled-release drug delivery systems^4^.

Additionally, Fig. 6a presents the percentage of Mg(OH)_2_ release in simulated gastric fluid with respect to its consumption in the release test. The Mg(OH)_2_ release percentages at 3, 6, 12, and 24 h are 1.5%, 3%, 6%, and 12%, respectively. The Mg(OH)_2_ release percentage in simulated gastric fluid versus time is modeled with a correlated model showing a slope of 0.5.

The robust and consistent release profile may be attributed to the effective encapsulation by okra mucilage, which forms a hydrophilic carbohydrate wall for the EMgPs, acting as a protective colloid for them. In line with the study by Kulshreshtha et al.^51^, carbohydrates, due to their hydrophilic attributes and biopolymeric macromolecule structure, provide steric stabilization for EMgPs, resulting in a sustained release of Mg(OH)_2_ NPs. The gel-like matrix of the mucilage acts as a stable diffusion barrier that minimizes variability between samples. While minor fluctuations are common in release studies due to experimental and environmental factors, the narrow error margins observed here reflect the reproducibility of the encapsulation process and the strong physicochemical interactions between magnesium hydroxide and the mucilage matrix. This results in a zero-order release kinetic profile with a constant rate, advantageous for maintaining therapeutic drug levels and improving patient compliance. The presence of minimal variability confirms the reliability of the EMgPs formulation and highlights the potential of okra mucilage as an effective encapsulating agent for controlled oral drug delivery.

To better simulate physiological conditions, a second release analysis extends the study to sequential gastric and intestinal phases. EMgPs are exposed to simulated gastric fluid (pH 2 with pepsin) for 2 h, then transferred to simulated intestinal fluid (SIF) for 48 h. This sequential release profile (Fig. 6b) shows a steady and linear release of Mg(OH)_2_ over time. After the 2-h gastric exposure, about 1.5 mg/mL is released, consistent with previous observations. Release in SIF increases gradually, reaching approximately 14.75 mg/mL after 48 h, confirming sustained drug liberation over an extended period.

Kinetic fitting of the release data across the phases reveals:

1. *Gastric Phase (0–2 h)*: Zero-order release (R^2^ = 1) with a rate constant of 0.75 mg mL^−1^ h^−1^, indicating a constant release rate independent of concentration and effective drug release control in acidic gastric conditions.
2. *Intestinal Phase (2–50 h)*: Continued zero-order (R^2^ = 0.9998) release with a reduced rate constant of 0.2894 mg mL^−1^ h^−1^, reflecting slower but steady drug release in intestinal fluid.
3. *Overall Release (0–50 h)*: The cumulative release, considering both phases, follows zero-order (R^2^ = 0.9978) kinetics with a rate constant of 0.2968 mg mL^−1^ h^−1^, underscoring the system’s ability to sustain controlled release throughout both digestive phases.

The findings highlight the potential of okra mucilage as an encapsulating agent in oral drug delivery systems and provide valuable insights for further research in the field of nanomedicine. The sustained release profile achieved through the use of okra mucilage could enhance the therapeutic efficacy of drugs, offering a more controlled and prolonged release, which is essential for maintaining optimal drug levels in the body and improving patient compliance. These results demonstrate the promising application of natural biopolymers like okra mucilage in the development of advanced drug delivery systems, paving the way for innovative treatments in nanomedicine.

## Conclusion

In conclusion, the study explored the utilization of nanoencapsulation technology, particularly employing okra mucilage as an encapsulating agent, for the development of magnesium hydroxide nano-capsules (EMgPs). Through a detailed fabrication process, the EMgPs were successfully synthesized, and their physicochemical and structural properties were comprehensively characterized using various techniques such as XRD, FTIR, Raman spectroscopy, and FESEM/EDS analysis. The results confirmed the complete encapsulation of magnesium hydroxide within the EMgPs, highlighting the potential of okra mucilage as a suitable encapsulating agent for oral drug delivery systems. The developed EMgPs exhibit favorable physicochemical properties and a hydrophilic nature, enhancing their solubility and stability in the digestive tract. Moreover, release tests conducted in a simulated digestive system environment demonstrated a controlled and sustained zero-order release profile of magnesium hydroxide from the EMgPs, indicating their potential for therapeutic applications. The findings underscore the promising role of natural materials, particularly okra mucilage, in the development of nanostructured drug delivery systems, offering a cost-effective and environmentally friendly alternative to synthetic encapsulating agents. Overall, the study contributes to the ongoing evolution of drug delivery systems, offering new avenues for the development of delivery systems loaded with bioactive compounds. Future research should focus on in vivo studies and explore other bioactive compounds as encapsulating agents using similar methodologies to fully exploit the potential of plant-based nanoencapsulation in pharmaceuticals.

## Data Availability

The data that support the findings of this study are available on request from the corresponding author.
